# Potential implications of shortened rotation length for forest birds, bryophytes, lichens and vascular plants: An example from southern Swedish production forests

**DOI:** 10.1371/journal.pone.0289835

**Published:** 2023-12-15

**Authors:** Lisa Petersson, Delphine Lariviere, Emma Holmström, Matts Lindbladh, Adam Felton

**Affiliations:** 1 Southern Swedish Forest Research Centre, Swedish University of Agricultural Sciences, Alnarp, Sweden; 2 The Forestry Research Institute of Sweden, (Skogforsk), Uppsala, Sweden; Technical University in Zvolen, SLOVAKIA

## Abstract

The rotation lengths of intensively managed production forests may be altered to achieve a variety of goals, with correspondingly implications for biodiversity. Here we consider the potential implications of shortened rotation times for biodiversity in planted monocultures of the two most common production tree species in Sweden, Scots pine (*Pinus sylvestris*) and Norway spruce (*Picea abies*). To do so we surveyed bird, bryophyte, epiphytic lichen and vascular plant diversity in 80 and 55-year-old stands; stand ages which approximate present-day and potential future rotation lengths in this region respectively. We found clear differences in the species communities of the 55 compared to the 80-year-old stands for both understory species and epiphytes, but not for birds. Nevertheless, bird species richness was still highest in the 80-year-old Norway spruce dominated stands. Dead wood amount was also highest the 80-year-old Norway spruce stands. Highest species richness of epiphytic lichens was found in 80-year-old Scots pine stands. However, 55-year-old Scots pine stands had a higher understory species richness and diversity than the older Scots pine stands, including a larger number of open land species. The 80-year-old forest stands examined may be considered old with respect to production forest rotation lengths in Sweden but are relatively young when comparing stand ages of unmanaged natural forest stands. Nevertheless, our results indicate that shortening the rotation time of Scots pine and Norway spruce, in this part of Sweden from 80 to 55 years, could have important consequences for forest biodiversity. These consequences are primarily inferred from the likely implications from shortened rotations for lichens community composition and diversity in both Norway spruce and Scots pine stands, as well as impacts on understory plant species in Norway spruce stands.

## Introduction

In Sweden, clear-cutting started becoming the dominant method for forest harvesting in the 1940s [1]. Today all but a small proportion of production forests are managed using clear-cutting with retention, and older natural and semi-natural forests continue to be replaced with intensive even-aged forestry [2]. This has negatively affected many forest species that depend on old natural forest habitats, contributing to the almost 2000 forest species on Sweden’s red-list [3]. As the vast majority of Sweden’s productive forest area is subject to such intensive forestry, and only a limited proportion of forest area has formal protection, each decision taken by production forest managers can have important implications for forest biodiversity [4]. A primary determinant of forest biodiversity in these even-aged stands is the rotation length, which is defined as the time period between two final fellings [5]. The rotation length dictates which forest structures will have time to develop within a stand [6], and how prevalent these structures will be throughout the production forest landscape [7].

In Sweden the optimal rotation length is mostly defined by economic indicators related to wood production, and dictated by the age when final felling will generate the maximum economic yield [5]. This principle of optimizing time of harvest, is often utilized by forest owners for the planning and prioritizing of management operations over larger properties [8]. The resultant outcome is then heavily influenced by site productivity and the choice of tree species; which is typically one of two native conifers, Norway spruce (*Picea abies*) and Scots pine (*Pinus sylvestris*), that together account for 79.4% of standing tree volume [9]. The rotation length is to some extent further constrained by Swedish law, which sets a minimum allowed stand age for final felling that varies with site productivity and species, but ranges from 45 years (e.g. fertile Norway spruce sites in southern Sweden) and 100 years (e.g. low productive Scots pine sites in northern Sweden) [10]. In southern Sweden, most Scots pine and Norway spruce stands on productive forestland (potential tree growth >1 m^3^ ha^−1^ year^−1^) are allowed to be clear-cut at an age between 50 and 65 years [11].

Today there are a number of potential drivers that act to reduce the rotation length, particularly in Norway spruce stands. These include more efficient forest management, improved growing material, and increased demand for biomass, as well as the desire to harvest stands before being damaged by wind, bark beetle (*Ips typographus*) or root rot (*Heterobasidion* spp.); risks that are all expected to increase under climate change [5, 12]. In contrast to Norway spruce, Scots pine production stands are not under the same pressure to reduce rotation lengths. This stems in part from the most prominent risk to Scots pine stands (browsing by large herbivores) occurring during stand establishment [13], and the high demand associated with large diameter Scots pine timber. The net result is that Scots pine stands are generally managed over longer rotation lengths than Norway spruce. However, there are three potential arguments for a change to shorter rotations also in Scots pine forestry; improved regeneration methods, a prolonged vegetation period due to climate change, and the use of genetically improved seedling material, can all contribute to faster growth and shorter rotations [14–16].

In this study, we examined the potential effect on forest biodiversity from shortened rotation times in Norway spruce and Scots pine stands. To assess the potential implications for forest biodiversity, we focused on four taxa with different life strategies and habitat requirements: birds, bryophytes, epiphytic lichens and vascular plants. By including such a diversity of taxa in one study, we are able to consider a number of questions regarding the potential consequences of reducing rotation times spanning a broader range of ecological responses. Specifically, we aimed to answer the following research questions: How could the implementation of shorter rotation length affect stand structure, species richness and species diversity for birds, bryophytes, lichens and vascular plants? Furthermore, how would it specifically affect red listed species? How would rotation length and tree species affect the community composition of birds, bryophytes, lichens and vascular plants? We also try to disentangle how the rotation length affects the species richness and diversity, and proportions of forest, generalist and open land species, respectively.

## Material and methods

We analysed empirical data from 20 Scots pine and 20 Norway spruce dominated production forest stands in southern Sweden. Stands in this area are typically managed using one to three thinnings during the rotation, starting with an initial planting density of 1500–3000 stems ha^-1^ which at time of final harvest would be reduced to 600–800 stems ha^-1^. To assess the potential impact of a shortened length, we used stands of 80 and 55 years. The lower stand age is set to match the lower baseline for when a stand is allowed to be harvested according to Swedish legislation (SFA 2020). The 80-year-old stands approximate the average age of stands being harvested in southern Sweden today (SFS 2018). By contrasting these stands with 55-year-old stands in the same region, we examined how communities of different taxa may be affected if rotation length is reduced.

### Study area

**Fig 1:**
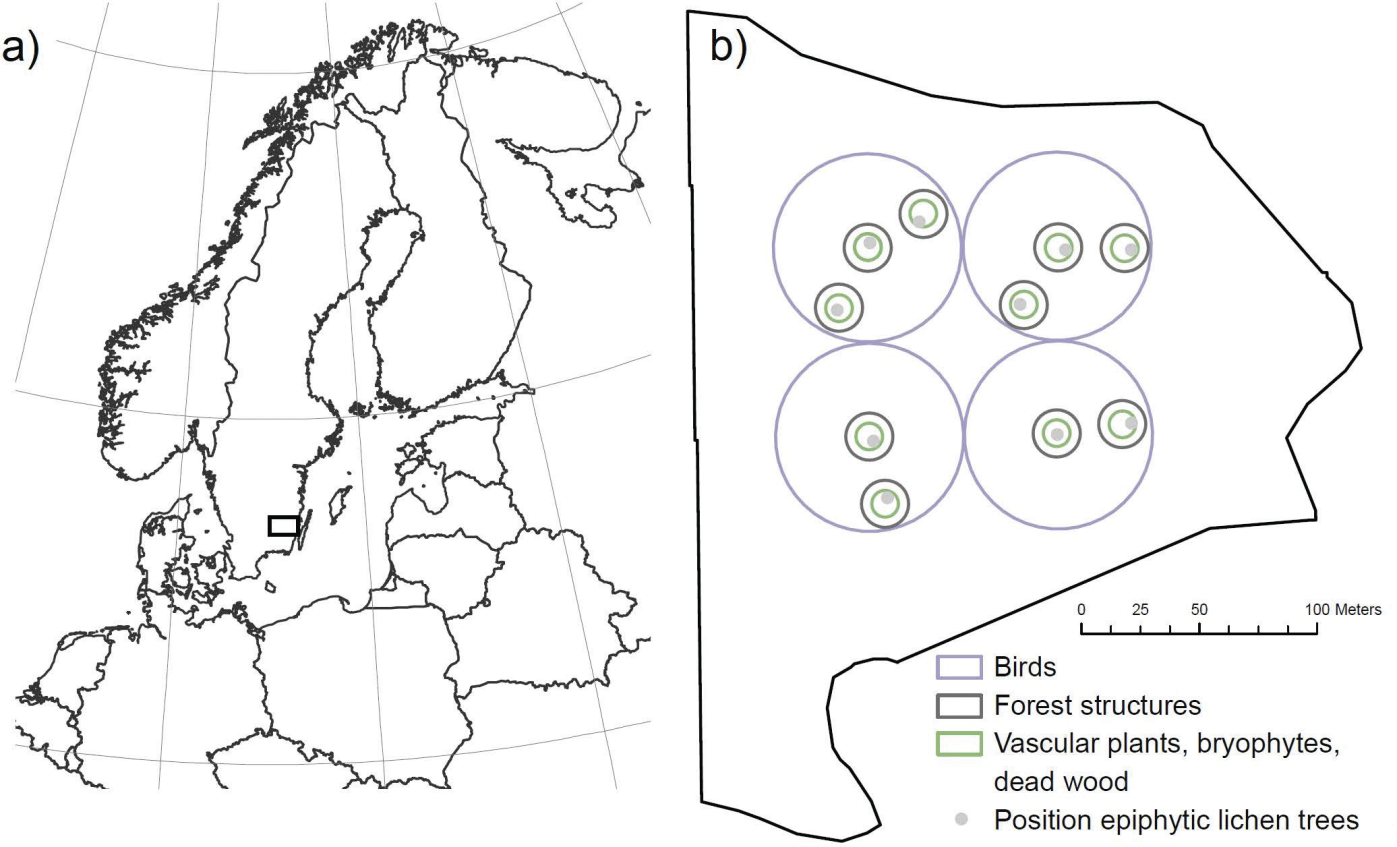
a) The study area is located in south-eastern Sweden. b) Survey data from forty Norway spruce and Scots pine dominated stands were used. In each stand, different sized areas were used for the surveying of different taxa. Four bird plots of 40 m radius (purple) were placed in the central parts of the stand. In the very centre of the bird plots, understory plots of 100 m^2^ (green) were placed. Another six understory vegetation plots were placed in random directions from the four central plots, resulting in 10 understory vegetation plots. In each of the ten plots, the crop tree (Norway spruce of Scots pine) closest located to the plot centre was selected for the epiphytic lichen survey (light-grey markers). Forest structures were measured in ten plots of 10 m radius (dark-grey). Made with Natural Earth. Free vector and raster map data @ naturalearthdata.com.

The study area was located in the hemiboreal zone [17] of southern Sweden (56°56′N, 15°34′E) (Fig 1A). In this region, the average growing season is 200–230 days and temperature ranges between 6–15°C in summer and (June–August) and down to -1°C in winter (December–February). Annual precipitation ranges from 800 mm in west to 600 mm in east [18]. Here standing volume of Scots pine and Norway spruce represent 29% and 49% of total volume respectively [19]. Common deciduous tree species are birch (*Betula pendula/B*. *pubescens*), oak (*Quercus robur/petraea*), aspen (*Populus tremula*), rowan (*Sorbus aucuparia*) and black alder (*Alnus glutinosa*), all of which may occur in stands as natural regeneration [20].

### Stand selection

In total 40 stands were selected, evenly distributed between Scots pine (20) and Norway spruce (20) dominated (>80% of basal area) stands. The stands consisted to two age categories: 55-year-old (±5 years) and 80-year-old (±5 years). To minimize variation due to site fertility, stand selection was concentrated to finding stands on intermediate fertile soils, i.e. where both Norway spruce and Scots pine typically is used as a crop tree. For that reason, forest plan site index (SI) was used for restricting stand selection. The SI is a common tool for measuring and comparing forest site productivity in forestry, and equals the projected dominant stand height in meters after 100 years of growth. Here, SI was restricted to 24–29 for Norway spruce. The corresponding SI value for Scots pine, was determined using a transformation formula [21]. To further minimize edaphic variation between stands, we only considered stands on till soils, with either rhyolite or granite bedrock (SGU, bedrock map, soil type map 1:25 000–1:100 000). In this part of Sweden, forest stands often occur on land that at previous time were used as pastures, meadows and crop fields. To minimize variation due to differences in previous land use, we used historical maps (Swedish land survey: Ekonomiska kartan 1941–1949) to select 55-year-old stands that were at least second generation of forest. This could not be done for the 80-year-old stands, because of insufficient historical maps from that time period.

### Species inventories

Birds were surveyed four times during spring: twice in late March–early April, and twice in late May in 2016. The periods were chosen to match the annual peak of singing activities, and to capture both resident and passerine bird species. Surveys were conducted at approximately 6:00–9:00 a.m. in early spring and 4:30–7:30 a.m. in late spring. To minimize variation in bird activity and influences on detectability, surveys were only conducted in suitable weather conditions (i.e. minimal wind and no precipitation). Four 40 m radius circular plots were placed centrally in the stands (Fig 1B), from which point counts were conducted (Bibby et al. 2000 [22]), with each survey lasting five minutes. The centralized location of plots was done to reduce the influence of birds inhabiting the forest edge. Plots were also centralized to reduce the effect of surveying over different spatial extents in larger versus smaller sized stands. The location of the four survey points was decided beforehand using a geographic information system (GIS) (see Lindbladh, Petersson [23] for details).

Understory vegetation was surveyed June-October in 2016. Forest floor bryophytes and vascular plants were surveyed on all types of substrates (i.e. soil, stone and dead wood) in 100 m^2^ circular plots, using a total of ten plots in each stand. The first four initial plots were placed in the very centre of the four bird plots (Fig 1B). After that, six additional plots were placed in random directions from these. To avoid edge effects, the plots were placed > 25 meters from the stand edge, and never closer to each other than 25 meters. See Petersson, Nilsson [24] and Petersson, Holmström [25] for more details about the understory vegetation inventory.

Epiphytic lichens were surveyed June–October 2017. In the centre of each of the ten circular plots (Fig 1B), the closest living crop tree was selected for the lichen survey. The presence/absence of epiphytic lichen species for each tree was recorded on the trunk and branches, up to two meters (see Petersson, Lariviere [26] for details).

### Stand structure measurements

Forest structures were measured in 2016 and 2017 in ten plots per stand (Fig 1B). Plot data was then averaged at the stand level. Dead wood (Ø > 10 cm) was measured in the same 100 m^2^ plots as used for the understory vegetation inventory. Basal area and stem densities were also measured in the same plot as were used for the understory vegetation survey, but here the measured area was extended to a radius of 10 m. However, in 80-year-old Scots pine stands where the number of production trees was low (< 5 trees within the 10 m radius), the area measured was extended to a 15 m radius. Canopy cover was calculated from hemispherical photos taken from the central point of the 100 m^2^ plots, at 1 m height, using a Nikon 5300 and a Sigma fisheye lense 4.5 mm 1:2.8. After excluding the two outer rings of the circular grid to avoid the inclusion of ground vegetation, pictures were analysed using Gap Light Analyzer [27].

The field work for the study was conducted on private owned forest land and land owned by Swedish state-owned forest company Sveaskog. No permissions were needed because the Swedish "Right of Public Access law" allows everyone to visit forests in Sweden.

## Statistical methods

We used Welch two sample t-test in R version 4.1.0 [28] to test differences between forest structures, species richness, diversity and the differences between number of forest, generalist and open land species.

### Species richness and diversity

We calculated species richness (number of species) and Shannon index (H) at three spatial levels (plot/tree, stand, landscape) for all taxonomic groups together (All taxa), and for each taxon individually (birds, bryophytes, lichens and vascular plants). For each stand category we plotted an accumulation curve to get an overview of species diversity only at stand level.

Shannon index (H) was calculated using the ‘diversity’ function from R package v*egan* [29] at three spatial levels (plot/tree, stand, landscape) according to the species list. Shannon index (H) weights species according to their frequency without considering their expected abundance [30]. “H” increases as the number of species increase in an assemblage, and with evenness of their distribution. Stand level Shannon indexes were then compared between age categories (55/80) for each tree species and taxa with a t-test. Because the bird survey was made in four plots (instead of ten) bird species richness and diversity was only calculated at stand and landscape level. Plot level Shannon indexes were compared between age categories for each tree species and taxa using a linear mixed model fit by REML, and t-tests comparison using the Satterthwaite’s method and “LmerTest” package [31]. The model included Shannon index per plot as the dependent variable, age as the explanatory variable, and stand as a nested random factor for each category. P-values were obtained from a Type II Wald chi-square test.

### Species accumulation curves

To produce species accumulation curves, we used the species incidence list (presence-absence) at stand level and for each taxon separately (birds, bryophytes, lichens and vascular plants). In total four groups were investigated, (Pine 55, Pine 80, Spruce 55 and Spruce 80). At stand level each species can have a maximum of 10 incidences, meaning that the species appears in every single stand of that category.

We produced species accumulation curves for each taxa and age/tree species category using the “iNEXT” R package [32]. We then obtained estimated diversity by Hill number, species richness and Shannon diversity for each category based on the interpretation of the species list from the 10 stands for each category. The ten plots per stand were used as the independent variable and each taxon diversity index (species number) was the dependent variable.

### Venn diagram

The distribution of unique species in different stand age categories was compiled and presented in a Venn diagram. We used the package ‘ggvenn’ [33] for producing the plots in R.

### Multivariate testing

To test for differences in community compositions between 55 and 80-year-old stands, among the different taxa, we used a permutational multivariate analysis of variance (permanova). The number of stands in which the species was registered (1–10) was used as a measurement of abundance in the multivariate analysis. The test was run in vegan R-package ‘adonis’ with 999 permutations [29]. Each taxon was tested separately for Norway spruce and Scots pine and stand age was set as an explanatory variable in the model.

### Categorization of forest, open land and generalist taxa

Birds, bryophytes, lichens and vascular plants species were categorized according to their association with forest or open land environments, with these in-turn defined as ‘habitat categories’ (Table 1). We used descriptions in Birds of the World (https://birdsoftheworld.org/bow/home) for bird species preferences for open- and forest land. For bryophytes, lichens and vascular plants, the classification was made according to Bernhardt-Römermann, Poschlod [34] Schmidt, Kriebitzsch [35] and Heinken, Diekmann [36]. Three species that were not found in the databases were categorized as open land species (*Vicia hirsuta*, *Carex oederi* and *Racomitrium lanuginosum*). The lichen *Cladonia chlorophaea* was categorized as a forest species. Four vascular plant species and genera could not be categorized and were hence excluded from this analysis (*Hieracium* sect. *hieracium*, *Malus domestica*, *Rosa* sp., *Carex* sp.). The two categories closed-and open forests (1.1 and 1.2) were combined here into one category ‘Forest’. Taxon with indifferent requirements when it comes to forest or open land were classified as ‘Generalist’.

**Table 1:** Short forms and explainations to the different habitat categories/forest affinity used in the article.

Shortform	Explaination
Forest	Prefers forests, glades and forest edges
Open land	Prefers open land, but may occasionally occur in forest
Generalist*	Occurs across closed forest and open land

*generalist in the terms of having no preference when it comes to shaded and open environments.

## Results

### Stand structures

The largest amount of dead wood was found in 80-year-old Norway spruce stands, which was significantly higher than that found in 55-year-old Norway spruce stands (Table 2). No difference was found between different aged Scots pine stands. Basal area was significantly higher in 80-year-old Scots pine stands but there was no difference in stem density between different aged stands. Norway spruce stands did not significantly differ in basal area nor stem density between 55 and 80-year-old stands. There was no significant difference in canopy cover between the different aged stands.

**Table 2:** Measured stand structures from the 40 Norway spruce and Scots pine stands. SD = standard deviation of the mean. Asterisks indicate significant structural differences between 55 and 80-year-old stands: *P <0.05, **P <0.01, ***P<0.001.

Tree species	Stand category	Dead wood (m^3^ ha^-1^)	SD	Basal area (m^2^ ha^-1^)	SD	Stem density (no ha^-1^) Ø> = 4cm	SD	Canopy cover (%)	SD
Norway spruce	55-year-old	6.2**	3.6	25.4	7.3	811	282	75.5	3.5
	80-year-old	22.4**	15.6	30.9	6.6	913	286	75.2	5.2
Scots pine	55-year-old	4.1	2.4	16.6**	2.8	651	139	53.1	4.7
	80-year-old	5.1	3.7	22.6**	4.2	611	362	52.0	7.1

### Species richness

**Fig 2:**
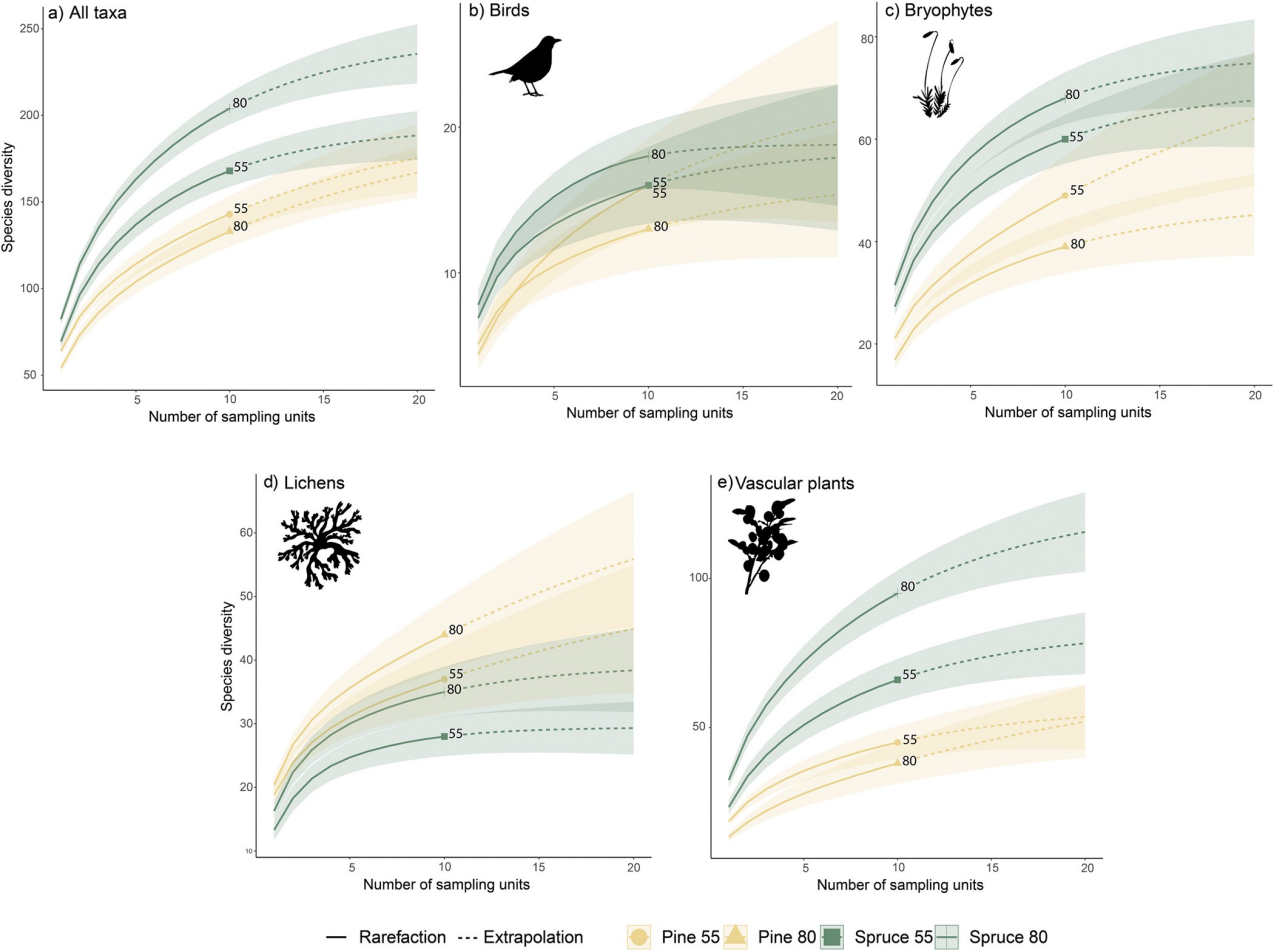
Coverage‐based rarefaction and extrapolation (R/E) sampling curves for each of the taxon a) all taxa pooled together, b) birds, c) bryophytes, d) lichens and e) vascular plants) at stand level based on Hill’s numbers qΔ with q  =  0 (species richness). The plots show the randomized interpolated accumulation curves (solid lines) for each of the ten 55 and 80-year-old Norway spruce and Scots pine stands. Results were extrapolated to double the amount of stand inventoried (dashed lines). Shaded areas indicate the 95% confidence intervals obtained using the bootstrap method on the basis of 100 repetitions.

We recorded a total of 270 different species in the forty stands. When considering the species richness of all taxonomic groups together, the highest total number of species was found in the 80-year-old Norway spruce stands (204), followed by the 55-year-old Norway spruce stands (168), 55-year-old Scots pine stands (143) and the 80-year-old Scots pine stands (133) (Fig 2A). The 80-year-old Norway spruce and Scots pine stands together supported 238 species, whereas 55-year-old Norway spruce and Scots pine stands together supported 203 species.

### Species diversity

When combining all species groups and attributing each stand a Shannon diversity, the mean Shannon diversity index between age classes for Norway spruce was marginally significantly different (p = 0.051). With 80-year-old Norway spruce scoring 4.4 ± 0.12 SD and 55-year-old Norway spruce scoring 4.21 ± 0.26 SD. For Scots pine, mean Shannon diversity per stand including all taxa together was significantly higher (p = 0.01) in 55- (4.15 ± 0.12 SD) compared to 80-year-old stands 3.98 ± 0.14 SD.

**Fig 3:**
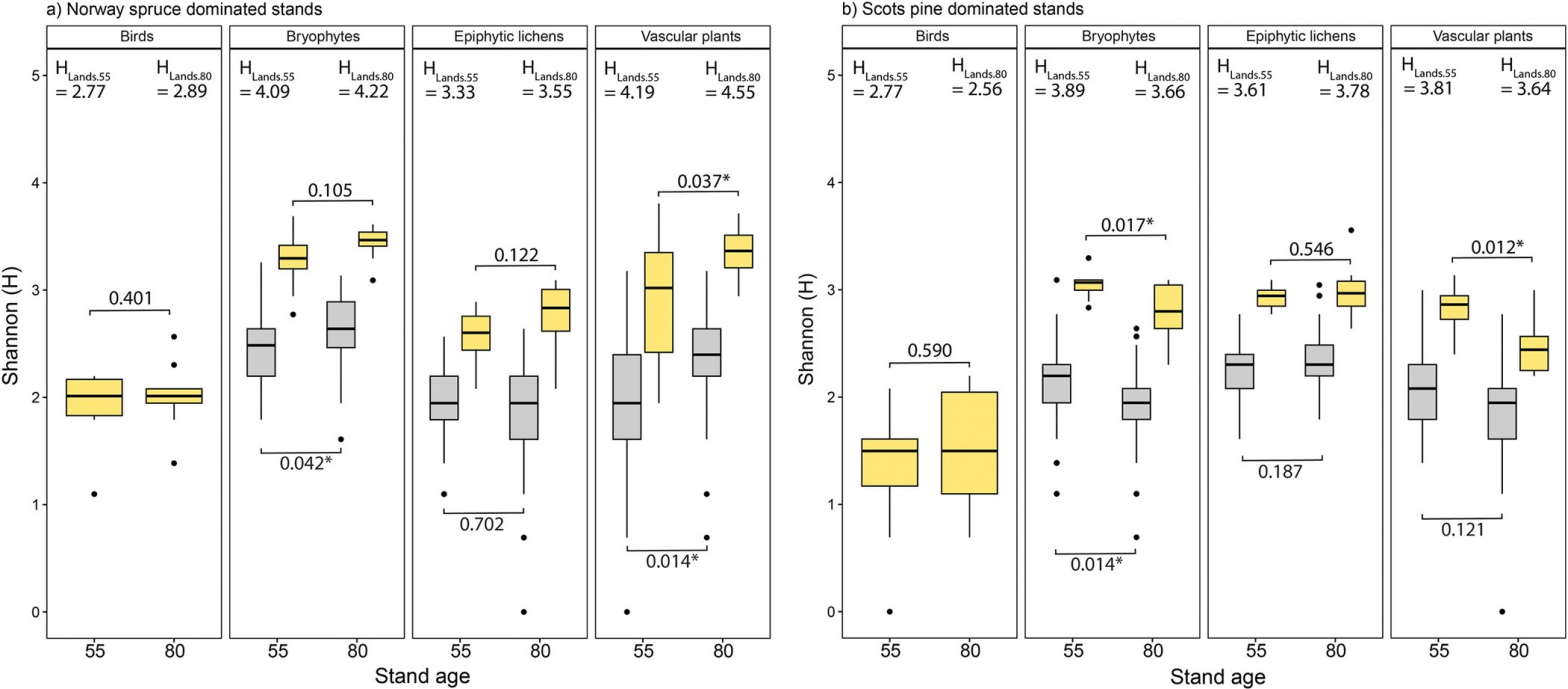
Shannon diversity index per stand (grey) or per plot (yellow) for each taxonomic group in stands dominated by a) Norway spruce and b) Scots pine. The landscape Shannon diversity index is calculated for each taxon as H_Lands.55_ for 55-year-old and H_Lands.80_ for 80-year-old stands. The boxplots show the distribution of the Shannon index (H) per plot or per stand and the black horizontal line shows the median value among age group in the data set. Whiskers above and below the boxes indicate the 10th and 90th percentiles. Points above and below the whiskers indicate outliers outside the 10th and 90th percentiles. P values are obtained from comparison of means via t-test for stand level and via LMER for plot level. Degree of significance is represented by a star symbol “*”. See S1 Appendix for complete table.

The total number of bird species recorded was 26. Species richness of birds was highest in 80 (18) and 55-year-old (16) Norway spruce stands followed by 55 (16) and 80-year-old Scots pine stands (13) (Fig 2B). However, there was no difference in Shannon diversity of birds between 55 and 80-year-old stands of Norway spruce, nor between 55 and 80-year-old stands of Scots pine (Fig 3A and 3B).

Total number of bryophyte species was 84. The highest Shannon diversity was found in 80-year-old Norway spruce stands (68) followed by 55-year-old Norway spruce stands (60), 55-year-old Scots pine (49) and 80-year-old Scots pine (39) (Fig 2C). In Norway spruce stands, Shannon diversity of bryophytes was significantly higher in older stands at the plot level, but there was no significant difference at stand level (Fig 3A). In Scots pine stands, bryophyte diversity was significantly higher in 55 compared to 80-year-old stands, at both stand and plot level (Fig 3B).

In total 57 lichen species were recorded. The highest lichen diversity was found in 80-year-old Scots pine stands (44) followed by 55-year-old Scots pine stands (37), 80-year-old (35) and 55-year-old (28) Norway spruce stands (Fig 2D). There was no difference in Shannon diversity of lichens between 55 and 80-year-old forest stands, neither for Norway spruce nor for Scots pine (Fig 3A and 3B).

In total 103 different vascular plant species were recorded. The highest species richness of vascular plants was found in 80-year-old Norway spruce stands (89), followed by 55-year-old Norway spruce stands (63), 55-year-old (41) and 80-year-old Scots pine stands (34) (Fig 2E). Vascular plant species diversity was significantly higher in 80 compared to 55-years-old Norway spruce stands at stand level (Fig 3A). In Scots pine stands, diversity of vascular plants at stand level was instead higher in 55 compared to 80-year-old stands (Fig 3B).

### Unique and red listed species

In total 100 species were exclusively encountered in 80-year-old stands. The highest number of unique species (species only occurring in either 80 or 55-year-old stands) was found in the 80-year-old Norway spruce stands (70) and the lowest number of unique species was found in 55-year-old Norway spruce stands (27). In the Scots pine stands, a higher number of unique species (41) were found in 55-year-old stands, compared to the 80-year-old stands (30). The 80-year-old stands of both Scots pine and Norway spruce had more unique red listed species (3) than 55-year-old stands (1).

**Fig 4:**
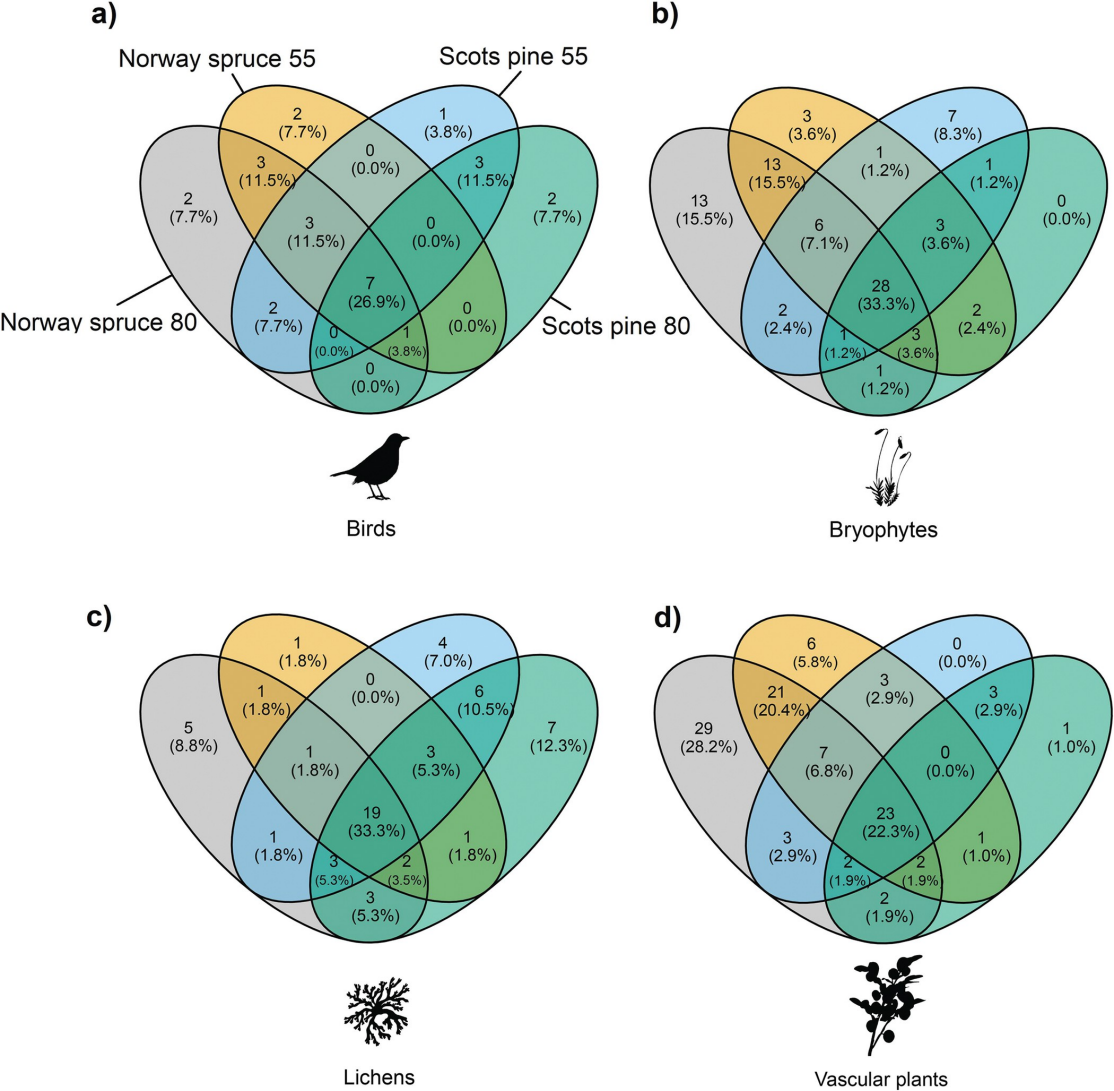
Venn diagrams showing shared and unique species of a) birds, b) bryophytes, c) lichens and d) vascular plants, when comparing 55 and 80-year-old stands of Norway spruce and Scots pine (as specified in panel a).

From the total number of bird species, 15.4% (7.7 + 7.7) were only recorded in 80-year-old stands and 11.5% were only recorded in 55-year-old stands (Fig 4A). Altogether, 26.9% of bird species were found in all stand types. For bryophytes, 15.5% were only recorded in 80-year-old Norway spruce stands, and no species was unique for 80-year-old Scots pine stands (Fig 4B). 11.9% were only found in 55-year-old stands and 33.3% were found in all stand types. For lichens, 21.1% of species were only found in 80-year-old stands, 8.8% in 55-year-old stands and 33.3% were found in all stand types (Fig 4C). For vascular plants, 29.2% were only found in 80-year-old stands, 5.8% in 55-year-old stands and 22.3% were recorded in all stand types (Fig 4D).

In total eight red-listed species were recorded through the survey [3, 37]. Three red-listed bird species were recorded, two of which were only recorded in 80-year-old stands: *Accipiter gentilis*, NT was recorded in an 80-year-old Scots pine stand, and *Phylloscopus sibilatrix*, NT was recorded in an 80-year-old Norway spruce stand. *Poecile montanus*, NT was recorded eighteen times and found in all stand types.

The bryophyte species *Splachnum ampullaceum* was found once in a 55-year-old and once in a 80-year-old Scots pine stand. The species is not red listed in Sweden, but is listed as near threatened (NT) in the IUCN Red List of Threatened Species [37]. Of the two red listed lichens found, the crustose species *Hertelidea botryosa* (NT) was encountered on two trunks in one 80-year-old Scots pine stand, whereas the fruticose filamentous species *Alectoria sarmentosa* (NT) was recorded once, on a branch of an 80-year-old Norway spruce. The red-listed vascular plants included *Scorzonera humilis* (NT), which was found once in a 55-year-old Scots pine stand and once in a 55-year-old Norway spruce stand. In addition, the orchid *Goodyera repens* (VU) was found once in an 80-year-old Scots pine stand and once in an 80-year-old Norway spruce stand.

### Community composition

There were significant (p<0.05) differences in community composition between 55-year-old and 80-year-old Scots pine stands for bryophytes and lichens (Table 3). For birds and vascular plants, the variation in communities between 55-year-old and 80-year-old stands was not significantly different from the variation among stands of the same age. For Norway spruce stands, communities of bryophytes, lichens and vascular plants were significantly different between 55-year-old and 80-year-old stands. For the bird communities, no significant difference occurred between communities in 55-year-old and 80-year-old stands.

**Table 3:** Differences in community composition between 55-year-old and 80-year-old Norway spruce and Scots pine monocultures. Community variances at stand level for birds, bryophytes, lichens and vascular plants were tested in permanova with 999 permutations in vegan package ‘adonis’. Significant results (p<0.05) are in bold.

	Norway spruce: 55-year-old– 80-year-old			Scots pine: 55-year-old– 80-year-old		
	F	R^2^	Pr (>F)	F	R^2^	Pr (>F)
Birds	1.66	0.08	0.14	1.50	0.08	0.22
Bryophytes	2.12	0.10	**0.031**	2.69	0.13	**0.005**
Lichens	4.56	0.20	**<0.001**	4.59	0.20	**<0.001**
Vascular plants	2.53	0.12	**0.014**	1.93	0.09	0.089

### Forest, open land and generalist species

At stand level (all taxa in all plots pooled together), the number of forest and generalists species were significantly higher in 80-year-old stands of Norway spruce, compared to 55-year-old stands (Table 4A). The number of open land species did not differ significantly. In Scots pine stands, the number of forest and generalist species was similar different aged Scots pine stands and the number of open land species was significantly higher in 55-year-old stands (Table 4B).

**Table 4:** Stand level differences in the number of forest-, open land- and generalist species between 55-year-old and 80-year-old a) Norway spruce and b) Scots pine monocultures. Before the analysis, all species from all taxa pooled together at stand level.

	a) Norway spruce			b) Scots pine		
	55-year-old stands	80-year-old stands	P-value	55-year-old stands	80-year-old stands	P-value
**Number of species**						
Forest	28.5	36.2	**0.022**	17.3	16.4	0.585
Open land	3.3	3.0	0.399	4.3	2.8	**0.026**
Generalist	36.6	45.8	**0.032**	38.8	35.2	0.072

**Fig 5:**
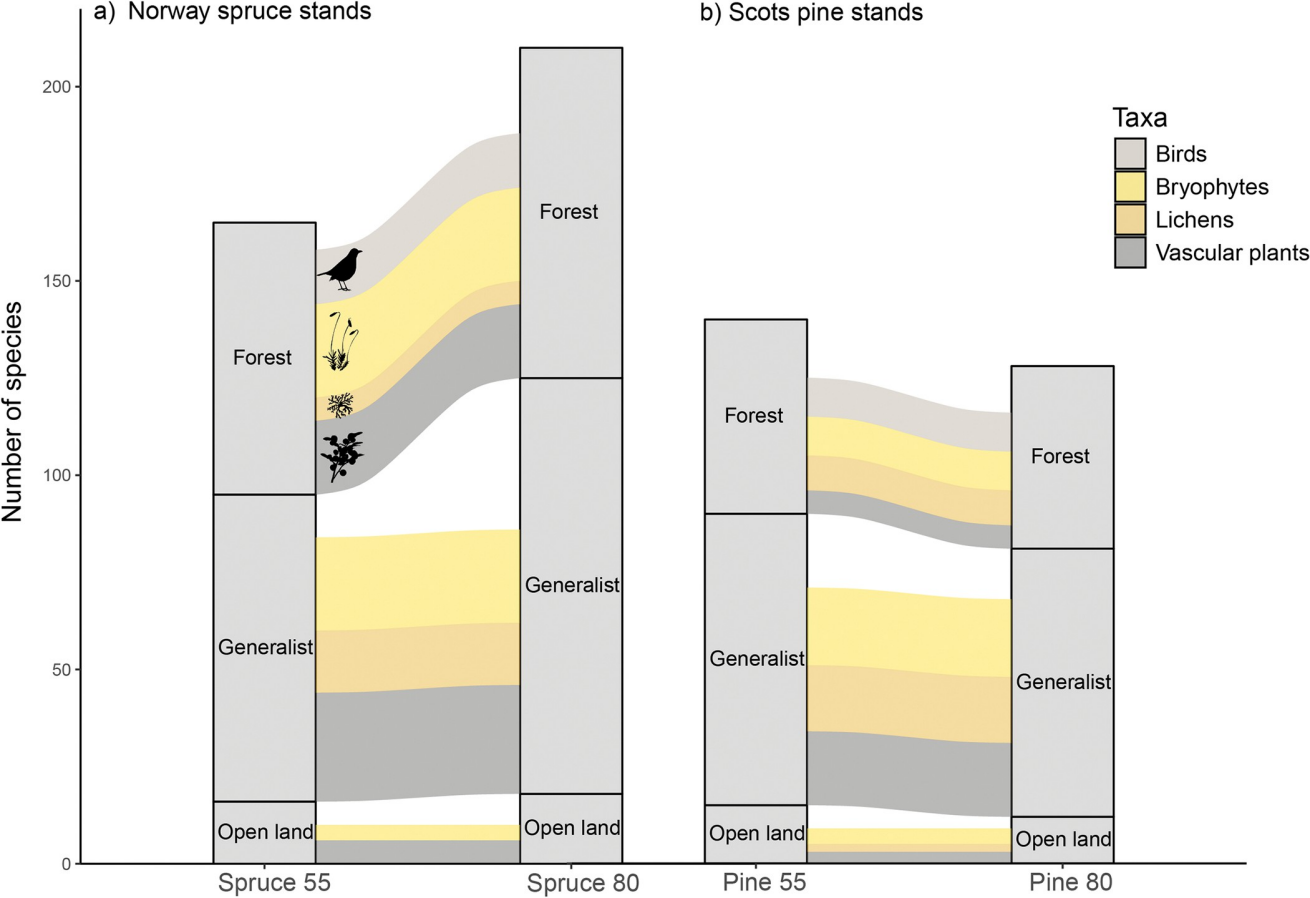
Proportion of different species groups at landscape level (all stands pooled together). Bars show the different aged stands and flows between bars illustrate the number of species occurring in both 55 and 80-year-old stands.

At the landscape level (pooling all stands and all taxa together), generalist species were the most common category of species (Fig 5). In both 55-year-old and 80-year-old Scots pine stands, 54% of the total number of species recorded were generalists. In Norway spruce stands, 48% of species were generalists in 55-year-old stands, and 51% in 80-year-old stands. Birds were the only taxon for which generalist species did not dominate. Instead, forest species was the most common category of birds (26), followed by generalist species (2). There were no open land species of birds observed. Forest species was the second most common group when considering all taxa together. The proportion of forest species was 36% in 55-year-old and 37% in 80-year-old Scots pine stands. In Norway spruce stands, the proportion of forest species was 42% in the 55-year-old stands and 40% in 80-year-old stands. Open land species had the lowest proportion of species, 11% in 55-year-old Scots pine stands and 9% in 80-year-old Scots pine stands. In 55-year-old and 80-year-old Norway spruce stands open land species constituted 10% and 9% respectively.

## Discussion

When examining 55 and 80-year-old monocultures of Norway spruce and Scots pine, we found several differences in stand structure, species richness and species communities. When it comes to stand structures, there was a large difference in proportions of dead wood between 55 (6.2 m^3^ ha^-1^) and 80-year-old Norway spruce stands (22.4 m^3^ ha^-1^) (Table 2). This was not the case in Scots pine stands, where the proportion of dead wood did not differ significantly between 55 (4.1 m^3^ ha^-1^) and 80-year-old stands (5.1 m^3^ ha^-1^). Species communities of bryophytes and lichens significantly differed between 55 and 80-year-old stands of both Norway spruce and Scots pine (Table 3). Vascular plant communities were also significantly different in the Norway spruce stand categories, however for bird communities, there was no difference between 55 and 80-year-old stands. We also found a significant increase of both generalist and forest associated species in 80-year-old Norway spruce stands compared to 55-year-old stands (Fig 5). For Scots pine stands however, there was no difference in number of forest and generalist species, instead the number of open land species was lower in 80-year-old stands. Overall, we found few red listed species in our stands. The number of red listed species was higher in 80-year-old stands; although this could not be tested statistically. These results indicate that shortened rotation times in this region of southern Sweden have the potential to influence both forest structures and biodiversity as a result. Below we discuss these results in the context of shorter rotation lengths and observed differences in select forest taxa between different aged stands. We also suggest how to adapt forest management practices for the promotion of biodiversity.

### Forest structures

Forest specialist microhabitats (i.e. old tree structures, richer supply and variety of dead wood substrates) often take long time to develop and are therefore more often associated with older forest stands. Many species of bryophytes are specialized on these old forest structures, e.g. dead trees, branches and stumps in different degree of decay [38, 39]. In addition, Norway spruce dead wood is an important substrate for a large number of fungi and insects [40, 41]. In this study, a relatively larger supply of dead wood was found in the 80-year-old Norway spruce stands compared to the other forest classes. The larger amount of ‘forest species’ and the larger richness and diversity of vascular plants and larger plot level diversity of bryophytes found in 80-year-old stands of Norway spruce may be in response. Here, a shortening of the rotation length e.g., by harvesting stands that are 55-years-old, could lead to both a loss of available dead wood substrates, a loss of forest associated species, and lower the species richness and diversity of understory taxa in these stands.

### Forest, open land and generalist species

Even though ‘forest species’ were found in all stand categories, generalists (in terms of openness) were more common and were the dominating type of species at stand- and landscape levels. One reason for this may be that species generalists are more tolerant to managed forest systems, where repeated thinnings and changing light conditions occur. Forest associated plant species are instead often favoured by more stable forest conditions [7] and may not tolerate rapid changes in light and humidity resulting from heavy thinnings and clear cutting [42–44]. The dominance of generalist species may also be explained by a large number of generalists in the available species pool. For instance, among vascular plants, the species pool for Swedish hemiboreal forests is dominated by generalist species in terms of light requirements [36].

In Scots pine stands there was no significant difference in the number of forest and generalist species between 55 and 80-year-old stands. However, the number of open land species was significantly higher in 55-year-old Scots pine stands. In addition, the diversity of bryophytes and vascular plants was also higher in 55-year-old Scots pine stands compared to 80-year-old stands. The reason for the decrease in open land species and the lower diversity of bryophytes and vascular plants in older Scots pine stands, may be because of the increasing dominance of a few competitive understory species in Scots pine stands over time. For example, the weft forming mosses *Pleurozium schreberi* and *Hylocomium splendens*, and dwarf shrub *Vaccinium myrtillus*, often dominate late successions of Scots pine forests on intermediate fertile sites in this area of Sweden. Consequently, when the understory layer remains undisturbed for a long time, as in 80-year-old Scots pine stands, these understory species may be able to outcompete other species and form an effective barrier preventing new species from establishing [45, 46]. This process may drive the resultant drop in total species richness, understory plant diversity, and open-land dependent species in the older Scots pine stands. In other words, the main difference that may explain the understory different response to stand age is that Scots pine stand understory communities are more likely to be determined by competition from understory plants, while Norway spruce understory communities are more likely driven by species accumulation.

### Community composition

Community composition significantly differed between the 80-year-old and 55-year-old stands for vascular plants, lichens and bryophytes, and thus for four of the five taxonomic groups assessed. In both Norway spruce and Scots pine stands, the community shift was most noticeable for epiphytic lichens (R^2^ = 0.2). In addition to the dependency on forest structural and climatic conditions, epiphytic lichens are also highly reliant on the substrate characteristic of their host tree. Typically, tree bark changes and more microhabitats develop as trees get older [47], which in turn may favour different types of lichen species than those using young tree bark. In addition, many lichen forest specialist species are dispersal limited [48]. This means that even if the conditions (substrate, micro climate) are right, they still may need long forest continuity before they are able to establish [49]. The particularly strong shift in epiphytic lichen communities in both Norway spruce and Scots pine stands shows the importance of preserving older trees to maintain diverse lichen populations.

Bird community composition did not significantly differ between 55 and 80-year-old stands, and there were also no significant differences in species richness and diversity between different aged stands. This result corresponds with earlier findings in the region [50]. One possible explanation is that important structures, in particular a broadleaved component, may have been removed in recurrent management actions, and that this management practice may have overruled the effect of stand age. However, a few species may nevertheless indicate a higher conservation value in the 80-year-old stands. For instance, great spotted woodpecker (*Dendrocopos major*) only occurred in 80-year-old Norway spruce stands, which may be explained by the higher volumes of dead wood in these stands. Another possible explanation for the lack of differences between bird communities of 55 and 80-year-old stands could be due to birds being mobile organisms. Their operation at larger landscape scales could in this case be overriding the effect of forest stand age in this study [23].

### Red listed species

The number of red-listed species encountered in the stands surveyed was too low to conduct meaningful statistics. Nevertheless, the highest number of red-listed species were associated with the older categories of Scots pine and Norway spruce stands respectively. The largest number of red listed species (5) was found in 80-year-old Scots pine stands followed by 80-year-old Norway spruce stands (4). Considering that many forest associated red listed species are associated with distinctive microhabitats often found in older and more natural-like forests than the stands in this study, the relatively low number of red listed species was not unexpected [51].

### Caveats and recommendations for future studies

In this study, we considered production forests for which stand age differed by 25 years and for which the upper age was 80 years old. Twenty-five years is a short time in a boreal forest perspective. Considering that trees of multi-layered Norway spruce stands under natural condition often can reach an age of more than 250 years—and over 170 years in Scots pine stands (example from north-western Russia) [52], the 80-year-old trees in this study are still biologically young. To fully understand the potential for biodiversity associated with hemiboreal Norway spruce and Scots pine forests, the inclusion of older stands in the study would have been needed.

Any intentional *a priori* shift in a stand’s intended rotational length would also be coupled to changes in the timing and number of thinnings (shorter rotations are generally coupled with fewer thinning events) [6]. As the stands used in this study were not intended for shorter rotations, our results are best seen as indicative of potential impacts on biodiversity. Also, we do not have information about past management (e.g. the number or timing of thinnings) or the establishment history of all of these stands. These are factors that may have affected the result. For example, some of the 80-year-old stands may have been established via natural regeneration on former agricultural lands. This establishment history may lead to differences in results in comparison to stands that were planted after clearcutting. Future research–that includes more taxa and preferably a wider variety of stand ages that extend in excess of 80 years of age–is needed to fully understand the consequences of changing rotation times.

### Conclusion

The result of this study shows the importance of considering several different taxa and metrics (e.g. species richness, diversity and community composition) when examining forest stand age effects on biodiversity. As the understory vegetation in Norway spruce stands seems to be primarily driven by species accumulation, bryophytes and vascular plant communities could also be strongly negatively affected from a shortening of rotation length in these stands. Scots pine understory communities did not seem to be driven by species accumulation but were instead determined by the dominance of a few species, mainly weft forming mosses and dwarf shrubs which may be driving a decrease of understory diversity in the older stands of this study. The effect of shortened rotation length would be notably negative for the epiphytic lichen communities, both in Norway spruce and Scots pine dominated stands. Because of the distinctively different lichen communities found in 55 and 80-year-old stands, we emphasize the importance of different stand ages, to maintain a rich epiphyte diversity. If Sweden is to maintain important habitats for the complete array of forest-dependent biodiversity in production forest stands, then our results indicate that shortened rotations would make it harder to achieve this goal. We draw this conclusion specifically from the likely implications from shortened rotations for lichens community composition and diversity in both Norway spruce and Scots pine stands, as well as impacts on understory plant species in Norway spruce stands.

## Supporting information

S1 Appendix Species richness and Shannon diversity index for each taxonomic group. (DOCX)
