# Supplementary material for: Potential implications of shortened rotation length for forest birds, bryophytes, lichens and vascular plants: An example from southern Swedish production forests
Source: PLoS One. 2023 Dec 15;18(12):e0289835. doi: 10.1371/journal.pone.0289835 (PMC10723730; doi:10.1371/journal.pone.0289835)
Supplement: S1 Appendix — (DOCX) [file pone.0289835.s001.docx]

Appendix 1

Table 1. Species richness in term of mean number of species (sppr) and Shannon diversity index for each taxonomic group, spatial scale and age classes for of a) Norway spruce and b) Scots pine. Differences in Shannon diversity between young and old stands are tested in a t-test at stand level and with a nested linear mixed model at plot level for birds, bryophytes, lichens and vascular plants.

a) Norway spruce stands

|  | **Level** | **Age** | **Sppr** | **SD** |  | **Shannon** | **SD** |  | **Lower.CL** | **upper.CL** | **SE** | **F value** | **P-value** |
| --- | --- | --- | --- | --- | --- | --- | --- | --- | --- | --- | --- | --- | --- |
| **Birds*** | **Stand** | **55** | 6,9 | 2,28 |  | 1,86 | 0,42 |  | 1,62 | 2,21 | 0,740 | 0,740 | 0,401 |
|  |  | **80** | 7,8 | 2,39 |  | 2,01 | 0,31 |  | 1,77 | 2,26 |  |  |  |
|  | **Landscape** | **55** | 16,0 | - |  | 2,77 | - |  |  |  |  |  |  |
|  |  | **80** | 18,0 | - |  | 2,89 | - |  |  |  |  |  |  |
| **Bryophytes** | **Plot** | **55** | 12,1 | 3,77 |  | 2,45 | 0,29 |  | 2,31 | 2,58 | 0,064 | 4,126 | **0,042** |
|  |  | **80** | 14,5 | 4,07 |  | 2,63 | 0,30 |  | 2,50 | 2,77 |  |  |  |
|  | **Stand** | **55** | 27,3 | 6,83 |  | 3,27 | 0,26 |  | 3,13 | 3,42 | 0,068 | 2,914 | 0,105 |
|  |  | **80** | 31,5 | 4,38 |  | 3,44 | 0,15 |  | 3,29 | 3,58 |  |  |  |
|  | **Landscape** | **55** | 60,0 | - |  | 4,09 | - |  |  |  |  |  |  |
|  |  | **80** | 68,0 | - |  | 4,22 | - |  |  |  |  |  |  |
| **Lichens*** | **Plot** | **55** | 7,2 | 2,16 |  | 1,92 | 0,32 |  | 1,76 | 2,08 | 0,077 | 0,147 | 0,702 |
|  |  | **80** | 7,1 | 2,55 |  | 1,88 | 0,42 |  | 1,72 | 2,04 |  |  |  |
|  | **Stand** | **55** | 13,3 | 3,47 |  | 2,55 | 0,29 |  | 0,04 | 0,85 | 1,310 | 2,628 | 0,122 |
|  |  | **80** | 16,3 | 4,71 |  | 2,75 | 0,33 |  | 0,92 | 1,09 |  |  |  |
|  | **Landscape** | **55** | 28,0 | - |  | 3,33 | - |  |  |  |  |  |  |
|  |  | **80** | 35,0 | - |  | 3,55 | - |  |  |  |  |  |  |
| **Vascular plants** | **Plot** | **55** | 8,2 | 4,62 |  | 1,95 | 0,57 |  | 1,70 | 2,20 | 0,120 | 6,025 | **0,014** |
|  |  | **80** | 11,6 | 4,65 |  | 2,37 | 0,43 |  | 2,12 | 2,62 |  |  |  |
|  | **Stand** | **55** | 23,3 | 12,60 |  | 2,89 | 0,63 |  | 2,57 | 3,20 | 0,150 | 5,054 | **0,037** |
|  |  | **80** | 32,4 | 8,15 |  | 3,36 | 0,23 |  | 3,05 | 3,68 |  |  |  |
|  | **Landscape** | **55** | 66,0 | - |  | 4,19 | - |  |  |  |  |  |  |
|  |  | **80** | 95,0 | - |  | 4,55 | - |  |  |  |  |  |  |
|  |  |  |  |  |  |  |  |  |  |  |  |  |  |

b) Scots pine stands

|  | **Level** | **Age** | **Sppr** | **SD** |  | **Shannon** | **SD** |  | **Lower.CL** | **upper.CL** | **SE** | **F or X2 value** | **P-value** |
| --- | --- | --- | --- | --- | --- | --- | --- | --- | --- | --- | --- | --- | --- |
| **Birds*** | **Stand** | **55** | 4,4 | 2,12 |  | 1,34 | 0,61 |  | 0,95 | 1,74 | 0,189 | 0,301 | 0,590 |
|  |  | **80** | 5,1 | 2,68 |  | 1,49 | 0,58 |  | 1,09 | 1,88 |  |  |  |
|  | **Landscape** | **55** | 16,0 | - |  | 2,77 | - |  |  |  |  |  |  |
|  |  | **80** | 13,0 | - |  | 2,56 | - |  |  |  |  |  |  |
| **Bryophytes** | **Plot** | **55** | 8,9 | 2,78 |  | 2,13 | 0,33 |  | 2,00 | 2,26 | 0,064 | 6,017 | **0,014** |
|  |  | **80** | 7,2 | 2,52 |  | 1,91 | 0,37 |  | 1,78 | 2,04 |  |  |  |
|  | **Stand** | **55** | 21,1 | 2,73 |  | 3,04 | 0,13 |  | 2,90 | 3,18 | 0,070 | 6,931 | **0,017** |
|  |  | **80** | 16,9 | 4,23 |  | 2,80 | 0,27 |  | 2,66 | 2,94 |  |  |  |
|  | **Landscape** | **55** | 49,0 | - |  | 3,89 | - |  |  |  |  |  |  |
|  |  | **80** | 39,0 | - |  | 3,66 | - |  |  |  |  |  |  |
| **Lichens*** | **Plot** | **55** | 9,8 | 2,13 |  | 2,26 | 0,22 |  | 2,17 | 2,36 | 0,045 | 1,745 | 0,187 |
|  |  | **80** | 10,7 | 2,69 |  | 2,35 | 0,23 |  | 2,25 | 2,44 |  |  |  |
|  | **Stand** | **55** | 18,8 | 2,10 |  | 2,93 | 0,11 |  | 2,80 | 3,06 | 0,060 | 0,380 | 0,546 |
|  |  | **80** | 20,4 | 5,93 |  | 2,98 | 0,26 |  | 2,85 | 3,12 |  |  |  |
|  | **Landscape** | **55** | 37,0 | - |  | 3,61 | - |  |  |  |  |  |  |
|  |  | **80** | 44,0 | - |  | 3,78 | - |  |  |  |  |  |  |
| **Vascular plants** | **Plot** | **55** | 8,3 | 2,97 |  | 2,05 | 0,36 |  | 1,89 | 2,22 | 0,081 | 2,399 | 0,121 |
|  |  | **80** | 7,0 | 2,46 |  | 1,88 | 0,39 |  | 1,71 | 2,05 |  |  |  |
|  | **Stand** | **55** | 18,5 | 4,30 |  | 2,82 | 0,26 |  | 2,64 | 2,99 | 0,080 | 7,885 | **0,012** |
|  |  | **80** | 13,3 | 4,16 |  | 2,48 | 0,27 |  | 2,31 | 2,66 |  |  |  |
|  | **Landscape** | **55** | 45,0 | - |  | 3,81 | - |  |  |  |  |  |  |
|  |  | **80** | 38,0 | - |  | 3,64 | - |  |  |  |  |  |  |
